# Targeted interplay between bacterial pathogens and host autophagy

**DOI:** 10.1080/15548627.2019.1590519

**Published:** 2019-03-25

**Authors:** Padhmanand Sudhakar, Anne-Claire Jacomin, Isabelle Hautefort, Siva Samavedam, Koorosh Fatemian, Eszter Ari, Leila Gul, Amanda Demeter, Emily Jones, Tamas Korcsmaros, Ioannis P. Nezis

**Affiliations:** a Earlham Institute, Norwich Research Park, Norwich, UK; b Gut Health and Microbes Programme, Quadram Institute, Norwich Research Park, Norwich, UK; c Department of Chronic Diseases, Metabolism and Ageing, KU Leuven, Leuven, Belgium; d School of Life Sciences, University of Warwick, Coventry, UK; e Current affiliation:Exaelements LTD, Coventry, UK; f Department of Genetics, Eotvos Lorand University, Budapest, Hungary; g Synthetic and System Biology Unit, Institute of Biochemistry, Biological Research Centre of the Hungarian Academy of Sciences, Szeged, Hungary

**Keywords:** Autophagy, bacterial regulation of host, MAP1LC3/LC3, MAP1LC3/LC3-interacting region motif, microbiota, CALCOCO2/NDP52, SQSTM1/p62, pathogen recognition, interplay

## Abstract

Due to the critical role played by autophagy in pathogen clearance, pathogens have developed diverse strategies to subvert it. Despite previous key findings of bacteria-autophagy interplay, asystems-level insight into selective targeting by the host and autophagy modulation by the pathogens is lacking. We predicted potential interactions between human autophagy proteins and effector proteins from 56 pathogenic bacterial species by identifying bacterial proteins predicted to have recognition motifs for selective autophagy receptors SQSTM1/p62, CALCOCO2/NDP52 and MAP1LC3/LC3. Using structure-based interaction prediction, we identified bacterial proteins capable to modify core autophagy components. Our analysis revealed that autophagy receptors in general potentially target mostly genus-specific proteins, and not those present in multiple genera. The complementarity between the predicted SQSTM1/p62 and CALCOCO2/NDP52 targets, which has been shown for *Salmonella*, *Listeria* and *Shigella*, could be observed across other pathogens. This complementarity potentially leaves the host more susceptible to chronic infections upon the mutation of autophagy receptors. Proteins derived from enterotoxigenic and non-toxigenic *Bacillus* outer membrane vesicles indicated that autophagy targets pathogenic proteins rather than non-pathogenic ones. We also observed apathogen-specific pattern as to which autophagy phase could be modulated by specific genera. We found intriguing examples of bacterial proteins that could modulate autophagy, and in turn being targeted by autophagy as ahost defense mechanism. We confirmed experimentally an interplay between a *Salmonella* protease, YhjJ and autophagy. Our comparative meta-analysis points out key commonalities and differences in how pathogens could affect autophagy and how autophagy potentially recognizes these pathogenic effectors.

**Abbreviations:** ATG5: autophagy related 5; CALCOCO2/NDP52: calcium binding and coiled-coil domain 2; GST: glutathione S-transferase; LIR: MAP1LC3/LC3-interacting region; MAP1LC3/LC3: microtubule associated protein 1 light chain 3 alpha; OMV: outer membrane vesicles; SQSTM1/p62: sequestosome 1; SCV: *Salmonella* containing vesicle; TECPR1: tectonin beta-propeller repeat containing 1; YhjJ: hypothetical zinc-protease.

## Introduction

Selective autophagy is a fundamental catabolic process where cytosolic material is specifically engulfed within double-membrane vesicles, known as autophagosomes, and targeted for degradation by lysosomes [1]. Xenophagy is a type of selective autophagy and refers to the selective autophagic degradation of invading bacteria and viruses, and is an important aspect of the host innate immune response to protect against infection [1–3]. Upon bacterial infection, autophagy is induced via multiple host factors and signaling pathways resulting in pathogen encapsulation within double-membrane structures called autophagosomes which ultimately fuse with lysosomes, causing the degradation of the enclosed bacteria [1,4–8].

Various proteins are involved in the autophagy process, with MAP1LC3/LC3 (microtubule associated protein 1 light chain 3 alpha) being among the most prominent [9,10]. MAP1LC3/LC3 is known to have a stable association with the autophagosome membrane, and can recruit cargo for degradation via a conserved LIR (LC3-Interacting Region) motif found in selective autophagy [11] receptor proteins such as SQSTM1/p62 (sequestosome 1), OPTN (optineurin) and CALCOCO2/NDP52 (calcium binding and coiled-coil domain 2) [12–14]. Besides their ability to bind autophagy receptors, MAP1LC3/LC3 proteins have also been shown to interact directly with proteins degraded by autophagy [15]. Pathogen-targeting by the autophagy machinery happens i) through direct protein-protein interactions between selective autophagy receptors and bacterial effector proteins [16–19] and ii) also occurs in an ubiquitin-dependent manner wherein the autophagy receptor interacts with and recognizes ubiquitinated cargoes [7,20]. Independently of canonical autophagy, MAP1LC3/LC3 can also be recruited to single-membrane phagosomes in a process called LAP (MAP1LC3/LC3-associated phagocytosis). This process does not depend on ubiquitination of the invading bacteria, but contributes to its degradation by the lysosomes [21].

Conversely, autophagy is often hijacked or manipulated by pathogenic bacteria, such as *Salmonella, Shigella* and *Helicobacter spp* [2,3,22,23].. Bacterial pathogens can either modulate their membrane surface, or express effectors and toxins to interfere with autophagy, and hijack autophagy to increase intracellular replication and further invasion potential [2,3,22,23]. Bacteria are able to avoid the autophagic defenses of the host through a variety of methods that can broadly be categorized into 3 different approaches – evasion, inhibition, and subversion. Bacterial evasion of host autophagy means escaping autophagic clearance or avoiding autophagic detection inside the cell. For example, although ATG5 (autophagy related 5) and TECPR1 (tectonin beta-propeller repeat containing 1) target *Shigella* to the autophagosome by binding the bacterial IcsA/VirG protein (Outer membrane protein IcsA autotransporter) [24], *Shigella* secretes IcsB (Virulence protein IcsB) which competitively binds to IcsA and therefore helps the pathogen avoid detection by the host autophagic machinery [25]. Inhibition of autophagy refers to bacteria that are able to arrest autophagy to some degree by interfering with the core autophagy machinery. For example, *Legionella pneumophila* infection in mammalian cells results in cell-wide cessation of autophagy. This ability is conferred by RavZ (*Legionella pneumophila* effector protein), which is a cysteine protease that localizes to phagophores involved in the early stages of autophagosome formation and cleaves MAP1LC3/LC3 family proteins conjugated to lipids [26]. Some other bacteria are able to subvert host cell autophagy to aid their own survival. *Coxiella burnetii* trafficks within a vacuole along the endosome-lysosome pathway in order to replicate in a low pH environment [27,28].

Even though there have been documented reports of specific bacterial pathogen proteins interfering with host proteins during particular phases of autophagy, it is not apparent if pathogens have adopted clearly devised strategies to hijack autophagy, for example by preferentially modulating specific phases to suit their survival and pathological niches. Likewise, a systemic view of which bacterial effectors can potentially modulate autophagy is lacking. To the best of our knowledge, no integrative study has yet been carried out to investigate whether those bacterial proteins that directly affect the host (including modulating autophagy) are specifically targeted by autophagy for degradation. A limited number of computational approaches have been carried out to estimate the bacteria-host interaction networks [29–33]. Scheidel and colleagues for example simulated the effect of *in silico* knock-outs of specific autophagy receptors such as CALCOCO2/NDP52, OPTN and TBK1 (TANK binding kinase 1) on the xenophagic capturing of individual pathogens such as *Salmonella* [29]. Budak and colleagues were able to identify key signaling proteins in the host-*Salmonella* interaction network by combining protein-protein interactions and phosphoproteomic data, demonstrating the efficacy of a computational analysis guided validation approach [30–33]. However, such studies were either confined to particular pathogens or were focused only on the unidirectional effects on the host without accounting for complex bidirectional interplays. Recently, Behrends and colleagues carried out a systemic analysis to map the ubiquitinome both in the host and in *Salmonella* to provide a global insight to host-pathogen interplay, specifically mediated by ubiquitination [34].

Motivated by these unanswered questions and to complement existing approaches, we systematically investigated the interplay between potential bacterial proteins and host autophagy. Here, we describe putative interactions between bacterial proteins targeted by autophagy via human orthologs (such as MAP1LC3/LC3) of Atg8-family proteins and the selective autophagy receptors SQSTM1/p62 and CALCOCO2/NDP52 in an ubiquitin-independent manner. Importantly, we provide experimental validation for one of them, the *Salmonella* protease, YhjJ. To understand the potential modulatory effects of bacterial effectors on host autophagy, we also identified specific bacterial proteins which are capable of being post-translationally modified by autophagy proteins. By combining the 2 analyses, we were able to identify a potential subset of bacterial proteins which could not only target autophagy but could also be targeted by autophagy. Specifically identifying the proteins targeted by autophagy receptors will provide a starting point for further study into how bacteria are marked for degradation and therefore how autophagy is triggered. This in turn will enable studies to improve the understanding of how bacteria behave during the course of an infection, how host cells respond, and if any of these mechanisms can be exploited for medical treatments. Comparative studies on different pathogens could identify evolutionarily conserved and strain-specific targeting patterns that may guide therapeutic development strategies.

## Results and discussion

### Autophagy targets genus-specific proteins

**Figure 1.**
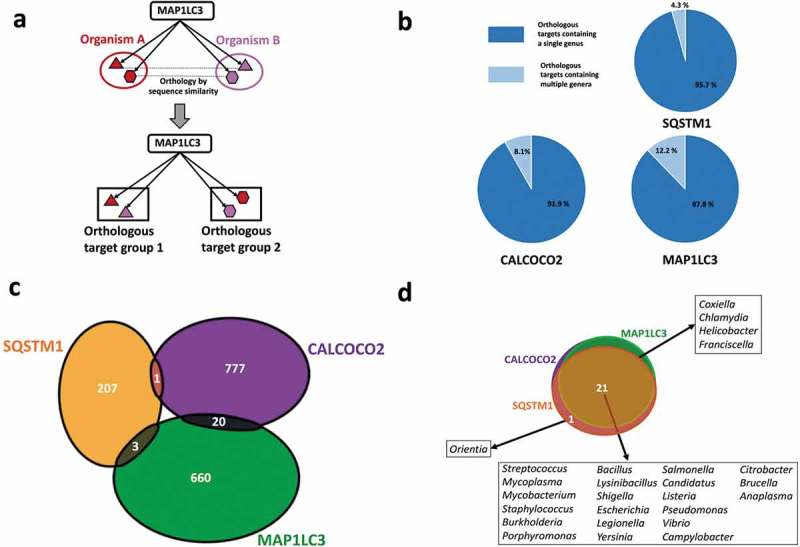
Genera and protein specificities of the autophagy receptors SQSTM1/p62, CALCOCO2/NDP52 and the autophagy adaptor protein MAP1LC3/LC3. (**a**) Definition of orthologous target groups. Orthologous targets are defined as the set of orthologous proteins which share sequence homology with each other and recognized as substrates by a particular autophagy targeting protein. (**b**) Number of orthologous target proteins of SQSTM1/p62, CALCOCO2/NDP52 and MAP1LC3/LC3 in single and multiple pathogen genera. (**c**) Comparison of bacterial proteins targeted by SQSTM1/p62, CALCOCO2/NDP52 and MAP1LC3/LC3 indicating that targeting of bacterial proteins by autophagy is mostly complementary. (**d**) Comparison of the studied bacterial genera targeted by SQSTM1/p62, CALCOCO2/NDP52 and MAP1LC3/LC3 showing a high overlap, which may promote efficient pathogen surveillance.

The bacterial proteins we analyzed belonged to 56 different strains (Table S1) from 26 distinct genera of bacterial pathogens with known associations with host autophagy. We grouped the bacterial proteins targeted by each of the autophagy targeting proteins (SQSTM1/p62, CALCOCO2/NDP52 or MAP1LC3/LC3) into orthology based clusters (i.e., orthologous targets – Table S2) to comparatively analyze the targeting features of autophagy (Figure 1(a), Table S3). A large proportion of the orthologous clusters potentially targeted by the autophagy proteins contained just 1 bacterial genus (Figure 1(b)) indicating that autophagy could be able to recognize proteins that are genus-specific, and not widespread among pathogens. For example, MAP1LC3/LC3 potentially targets ClfA (clumping factor A), which is uniquely found in *Staphylococcus aureus* strains in comparison to the other pathogens in our study. ClfAs are known to contribute to the virulence mechanisms of *S. aureus* by binding to human fibrinogen [35,36] and inhibiting phagocytosis [37]. This suggests autophagy has evolved to possibly target-specific proteins and functions that could be important for the invasive properties of specific pathogens. On the other hand, autophagy targeting proteins could also recognize conserved proteins from various genera of pathogens in a smaller fraction of orthologous groups (Figure 1(b)). An example for this more general targeting strategy are the MAP1LC3/LC3 targeted FlgL (flagellar hook protein) orthologs, present in multiple genera of pathogens. FlgL has a key role in motility, which is important for the systemic invasive properties of many bacterial pathogens [38].

### Complementarity of autophagy targeting proteins potentially increase infection risk upon mutation

Autophagy receptors SQSTM1/p62 and CALCOCO2/NDP52 act independently of each other as demonstrated by the almost extreme exclusivity of their targets (only 2 proteins are targeted by both SQSTM1/p62 and CALCOCO2/NDP52 (Figure 1(c)). This complementarity between SQSTM1/p62 and CALCOCO2/NDP52 has so far only been shown for proteins from *Salmonella, Listeria* and *Shigella* [4,39], whereas we substantially extended it for a very diverse set of pathogens, indicating that this mechanism could be in principle observed across genera. Furthermore, MAP1LC3/LC3 shared a very small number of overlapping targets with SQSTM1/p62 and CALCOCO2/NDP52, suggesting that these 3 autophagy proteins elicit their action in targeting bacterial proteins in an almost independent, complementary manner (Figure 1(c)). These observations suggest the existence of possible selection pressures, which may have influenced the specific recognition features of the autophagy receptors.

While the 3 autophagy proteins target bacterial proteins with little overlap, 21 out of the 26 bacterial genera in our study were commonly targeted by all these 3 autophagy receptors (Figure 1(d)). The remaining five genera were targeted by any 2 of the 3 autophagy proteins (Figure 1(d)). Given the complete lack of overlap among the targets of the 3 autophagy proteins, and the relatively large overlap in terms of the genera, this observation shows that autophagy could potentially target different sets of bacterial proteins from a same pathogen. The redundancy in targeting a same genera is beneficial for robust pathogen recognition, while the complementarity in the specific bacterial proteins is advantageous to increase the surveillance coverage of autophagy.

Nonetheless, the extreme complementarity of the autophagy targeting proteins could make the host more susceptible to chronic disorders and infections if the gene encoding one of the autophagy targeting proteins becomes mutated, and the autophagy system is overloaded or suffers other malfunctions. For example, various studies have highlighted the association between mutations in CALCOCO2/NDP52 and Crohn disease [40] which is an inflammatory bowel disease characterized by an altered gut microbiome. Accordingly, there is an increased vulnerability to infections by foodborne pathogens such as *Salmonella* and *Shigella* in the intestine of individuals with Crohn disease [4,39,40]. Given the complementarity among the autophagy targeting proteins, the compromised function of CALCOCO2/NDP52 could increase the risks associated with exposure to CALCOCO2/NDP52 targeted pathogens (e.g. *Salmonella, Shigella, Escherichia* etc). In addition, as mutations of several other autophagy genes, most importantly NOD2 (nucleotide binding oligomerization domain containing 2) [41] and ATG16L1 (autophagy related 16 like 1) [42,43] are associated with Crohn disease, the lack of proper CALCOCO2/NDP52 surveillance could potentially result in even less efficient bacterial degradation in this autophagy deficient background. Similarly, mutations of SQSTM1/p62 have been associated with Paget disease, a bone development related disease that often causes increased risk of infection, and with amyotrophic lateral sclerosis (ALS) causing episodes of lower respiratory tract infections [44,45]. Relevant to both ALS and our observation, the *Orientia* genus is one of the two genera whose proteins are uniquely recognized by SQSTM1/p62. *Orientia* has been detected in the lungs of patients with acute respiratory distress syndrome [46]. These observations could indicate that in the SQSTM1/p62 mutant background of ALS patients, the lack of *Orientia* spp. detection is capable of contributing to this pulmonary disease.

### Autophagy targets virulence factors

**Figure 2.**
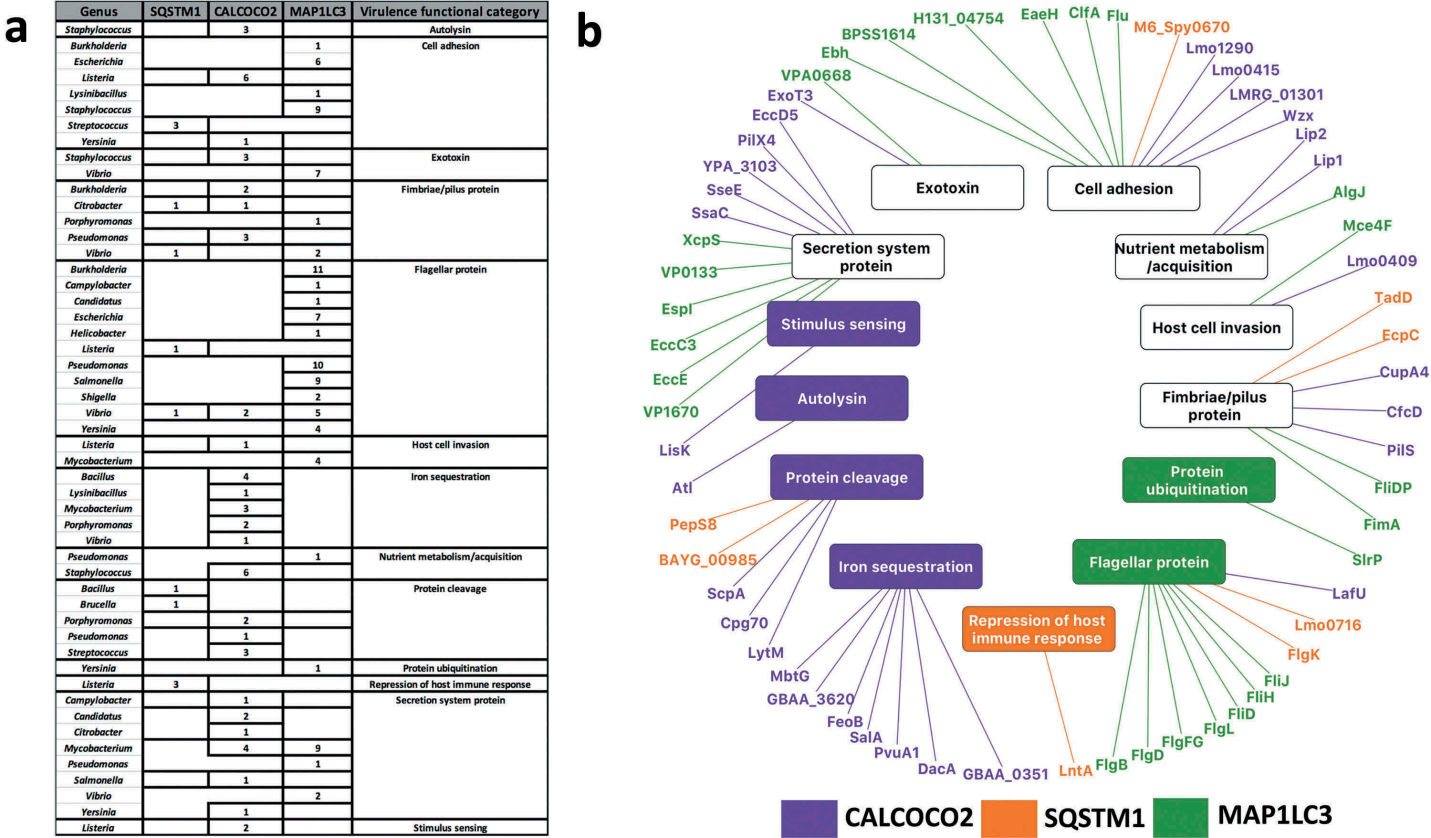
Virulence factor targeting features of SQSTM1/p62, CALCOCO2/NDP52 and MAP1LC3/LC3. (**a**) Tabular representation of the virulence factor classes targeted by the autophagy receptors and MAP1LC3/LC3. Only those bacterial genera where the targeted bacterial proteins are annotated as virulence factors are listed. (**b**) A network based view of the virulence factors (outer ring) and the corresponding virulence functions (inner ring) targeted by the autophagy proteins. We colored the virulence functions based on the associations between the autophagy targeting proteins and virulence factors. Whereas some of the virulence functions are targeted by only 1 particular autophagy targeting protein, other functions are targeted by multiple autophagy targeting proteins (in black).

Using data from the Virulence Factor Database [47], we observed that autophagy recognizes more virulence factors than would be expected by chance considering all the potential target proteins: i.e, virulence factors were overrepresented (hypergeometric test; P-value<0.05) among the targets of CALCOCO2/NDP52 (0.048) and MAP1LC3/LC3 (2.25e-09) but not in the predicted targetome of SQSTM1/p62. By adding known functions to the targeted virulence factors, and grouping them into 13 distinct categories, we identified commonalities and specificities among the autophagy targeting proteins (Figure 2(a-b); Table S3). Figure 2(b) represents this specific targeting of virulence categories as a network with nodes representing the interacting members and edges the interactions. Out of the 14 functional categories, five specialized virulence factors such as autolysins, and iron sequestering proteins which were potentially recognized uniquely by a single autophagy targeting protein considered in this study (Figure 2(a)). Autolysins which were specifically targeted by CALCOCO2/NDP52 alone have been known to play a major role in infection and contribution to virulence by controlling the bacterial peptidoglycan structure, thereby evading detection by innate immune system receptors such as NOD2 [48]. Conversely, virulence factors responsible for more general functions such as nutrient acquisition and motility (e.g. flagella, fimbriae and pili proteins) were potentially recognized by multiple autophagy targeting proteins (Figure 2(b)). Since most intracellular pathogens rely on motility within the host cell to either establish infection, evade detection or to migrate towards host cell regions with their preferred nutrient source, it is reasonable to expect that general virulence functions such as motility are potentially targeted commonly by all 3 proteins to boost the first line of host defense. Thus, by fine-tuning the recognition of their targets to include generalized as well as specific virulence functions, selective autophagy provides the host an efficient and robust defense mechanism against many pathogens.

We also checked if there is any underlying evolutionary selection pressure on the proteins capable of being targeted by autophagy and their functional relevance in terms of virulence and pathogenicity. To this end, we performed an additional analysis by comparing the sequences of non-pathogenic *E. coli* reference strain K12 to multiple *Salmonella* strain to identify single nucleotide polymorphic (SNP) mutations in the predicted LIR motifs in the *Salmonella* proteins. We identified 18 orthologous groups corresponding to *Salmonella* proteins harboring LIR motifs and having SNPs compared to *E. coli*. 14 of the 18 orthologous groups were associated with virulence functions such as protein cleavage, host cell invasion and iron sequestration (Table S4). This could indicate that the proteins capable of being targeted by autophagy are under selective evolutionary pressure driven by virulence and pathogenicity.

We also identified a key difference between the virulence factor targeting features of CALCOCO2/NDP52, MAP1LC3/LC3 and SQSTM1/p62. As CALCOCO2/NDP52 is already known to specifically potentially recognize ubiquitinated bacteria during xenophagy, and the presented analysis showed CALCOCO2/NDP52 and MAP1LC3/LC3 targets were enriched with virulence factors, it is possible that CALCOCO2/NDP52 and MAP1LC3/LC3 evolved specifically to target pathogens or pathogenic proteins for autophagic degradation [20,21]. On the other hand, SQSTM1/p62, conspicuous by the very small number of virulence factor related functions it targets, is mostly involved in other types of autophagy (such as the removal of internal ubiquitinated protein aggregates). This may suggest that whilst SQSTM1/p62 could target more generic bacterial proteins containing a target motif but not (yet) related to virulence, CALCOCO2/NDP52 and MAP1LC3/LC3 probably evolved to exploit the particular niche of bacterial proteins that SQSTM1/p62 not capable of interacting with, thereby attenuating and improving the host’s ability to recognize and remove virulent molecules via autophagy.

To confirm that our observations were not biased by selecting pathogenic bacteria and only the proteins thereof which are accessible to the autophagy targeting machinery, we performed a control analysis with proteins found within bacterial outer membrane vesicles (OMVs) using the same workflow. OMVs are extracellular bodies [49] secreted by pathogenic [50–52] and non-pathogenic bacterial species including probiotics and commensals [53–55]. OMVs are engulfed by host cells upon endocytosis and due to this mode of action, the OMV contents including proteins can be spatially accessed by the host machinery [54]. We used a recent study [56], which listed the proteins from OMVs of pathogenic (enterotoxigenic) as well as non-pathogenic (nontoxigenic) strains of *Bacillus fragilis*. From the analysis, we found an overrepresentation of autophagy targeted proteins: from 140 enterotoxigenic OMV proteins 33 could potentially be targeted by the autophagy receptors SQSTM1/p62, CALCOCO2/NDP52 and the autophagy adaptor protein MAP1LC3/LC3 (hypergeometric test P-value 0.039) (Table S5). The results from this control analysis using pathogenic and non-pathogenic bacterial proteins known to be localized in the host cytosol and potentially affect the host processes including autophagy suggest that pathogenic proteins could be selectively targeted by autophagy to a greater extent than non-pathogenic proteins.

### Phase-specific regulation of autophagy by pathogens

**Figure 3.**
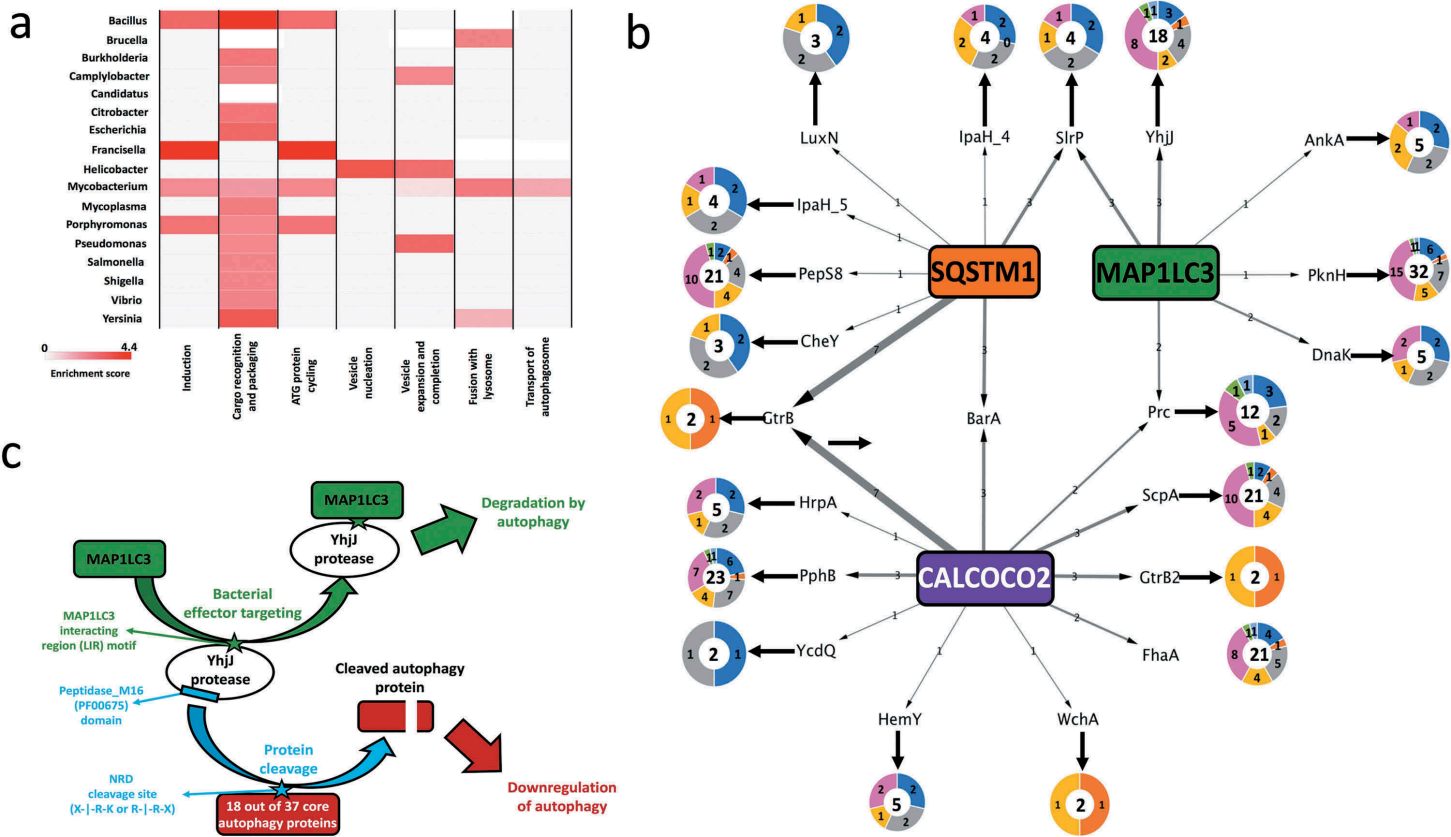
Pathogenic modulation of autophagy and the bi-directional interplay (**a**) Heat map showing the phase-specific regulation of autophagy by various bacterial genera. Hypergeometric distribution was used to determine the over-representation of proteins from each bacterial genus in our study and show those predicted (based on domain-domain and domain-motif interactions) to modulate proteins functioning in 1 or multiple phases of the core autophagy process. The significance score is determined as the -log10 function of the corrected hypergeometric distribution based enrichment P-value. (**b**) Interplay between autophagy receptors and their target bacterial effectors, which regulate different phases of autophagy. The donut plots display the phase classification of the core autophagy proteins targeted by each bacterial effector protein, and the total number of host autophagy proteins targeted by the bacterial effector is indicated by the bold number within the donut plot. The thickness of the arrows from SQSTM1/p62, CALCOCO2/NDP52 and MAP1LC3/LC3 denote the number of orthologs of the targeted bacterial effector. (**c**) An example of the interplay between host autophagy and the protease YhjJ from *Salmonella typhimurium SL1344.*

The previous sections outlined how autophagy targets bacterial effector proteins. But this is only one side of the story as bacterial proteins from various pathogenic genera are also potentially able to modulate autophagy [2,3,22,23]. We observed genus- specific patterns as to which phases of autophagy (e.g., induction, autophagosome formation, fusion with lysosome) are potentially regulated by a given pathogen group (Figure 3(a)). For example, from the over-representation analysis of bacterial proteins predicted to modulate autophagy proteins (Table S6), we observed that *Bacillus spp*. have propensity to putatively modulate multiple early phases of autophagy (‘Induction’, ‘Cargo recognition and packaging’ and ‘ATG protein cycling’ phases) than the further downstream phases. This ties in well with previous observations that *Bacillus anthracis* inhibits autophagy initiation *via* a cyclic AMP concentration-promoting adenylyl cyclase [57]. Our analysis suggests that the *Bacillus* genus also potentially modulates the autophagy induction phase by deploying effectors such as extracellular proteases and serine-threonine kinases to modify the post-translational states of key autophagy proteins, and subvert host defenses.

We noted that some autophagy phases could uniquely be modulated by particular pathogens (e.g., the ‘autophagosome-lysosome fusion’ phase uniquely by *Mycobacterial, Bacillus and Yersinia* species only), while some phases were modulated by multiple pathogen genera (e.g., the ‘Cargo recognition and packaging’ phase) (Figure 3(b)). Even though it is known that *Mycobacterial* species such as *M. tuberculosis* H37v accumulate in non-acidic or non-degradative autophagosomes during infection [58], the actual mechanisms responsible for this modulatory relationship have not yet been explained. From our computational analysis, we observed that multiple *Mycobacterial* species could potentially interfere and block the fusion of the autophagosome and lysosome *via* the action of various phosphate-group removing hydrolases as well as peptidases on TECPR1, which facilitates autophagosome maturation and autophagosome-lysosome fusion [59]. In mice, TECPR1 is a binding partner for ATG5 and colocalizes with ATG5 at *Shigella*-containing phagophores [24]. TECPR1 activity was also specifically found to be necessary for selective autophagy targeting bacteria and not for the more generic stress induced autophagy [24]. In this context, it is intriguing that intracellular pathogens such as those from the *Mycobacterial* genus could putatively regulate selective autophagy proteins like TECPR1, which is needed for the maturation of the autophagosome and the autophagosome-lysosome fusion [59]. The modulation of TECPR1 as inferred by our analysis provides clues that explains the underlying mechanisms contributing to the *Mycobacterial* induced blockage of autophagosome-lysosome fusion.

### Autophagy strikes back

To investigate co-evolutionary, interplay strategies, we looked for the overlap between bacterial proteins predicted to targeted by autophagy and autophagy proteins potentially modulated by the same bacterial protein. We were able to identify 67 such bacterial proteins (out of the 2119 bacterial proteins predicted to modulate autophagy) (Figure 3(b); Table S7). The low number of such special proteins is expected given the complex and co-evolutionary processes required to meet the strict selection criteria we applied. In support of this, we found that the 67 proteins were derived from 18 bacterial genera (out of the 26 examined), showing a fairly widespread strategy with a notable level of conservation (the 67 proteins were grouped into 40 orthologous groups). Most of the bacterial-autophagy interplays involved the recognizing function of either CALCOCO2/NDP52 or MAP1LC3/LC3. Analyzing the functions of the interplay-related bacterial proteins, compared to all the proteins targeted by autophagy in our study, indicated that the former has unique characteristics. Some of the interplay-related bacterial proteins have functions specifically related to proteolysis (YhjJ, ScpA [C5a peptidase]), phosphorylation (PknH [Serine/threonine-protein kinase PknH], PphB [Serine/threonine-protein phosphatase 2]), and post-translational effects such as transfer of ubiquitin (SlrP [E3 ubiquitin-protein ligase]) moieties, signifying that they are capable of successfully rewiring the host autophagy mechanisms. Due to their potential modulatory effects on autophagy, the interplay-related proteins could in turn be selectively targeted by autophagy for degradation as a counter-measure. 23 of the 40 orthologous groups represented proteins with proteolytic and post-translational activity such as phosphorylation and ubiquitination which can interfere with the activity of autophagy proteins, and thereby promote autophagy inhibition as shown previously, for example with the MAP1LC3/LC3 degrading function of the *Legionella* effector protease RavZ [26]. We also observed that as many as 7 of the orthologous groups involved in the interplay could potentially modulate a substantial portion of the autophagy machinery (i.e., at least 16 of the 37 core autophagy proteins). Thus, by targeting the bacterial effectors, which are capable of modulating a major part of the autophagy machinery, autophagy protects itself from these effectors as well as strengthening the overall surveillance in the host.

A relevant new example of a bacterial effector involved in the bacteria-autophagy interplay is YhjJ, which is a zinc-protease from the food-borne pathogen *Salmonella enterica* serovar Typhimurium SL1344 and was found to be expressed under conditions when *Salmonella* infects host cells [60]. YhjJ has an M16 peptidase domain (PFAM ID: PF00675), which can recognize the N-Arg dibasic (NRD) convertase cleavage site (X-|-R-K or R-|-R-X). We found this NRD cleavage site to be present in multiple core autophagy proteins distributed across all the seven phases of autophagy. Compared to the proteins in the whole human proteome harboring the NRD convertase cleavage site, we found a significant enrichment of core autophagy proteins possessing this cleavage site (hypergeometric distribution P-value 7.7E-03). The significant enrichment means that YhjJ could have a specificity with its M16 peptidase domain in acting on autophagy proteins. The M16 peptidase domain of YhjJ is similar to the M16 peptidase domain of insulysin and nardilysin and other metallopeptidases found in different bacteria and in eukaryotic parasite species to have protease activity on host proteins [61,62]. Nardilysin in *Helicobacter felis* for example regulates gastric inflammation upon infection by cleaving host proteins on their NRD cleavage sites confirming the physiological role of this interaction [63]. In agreement with the potential harmful role of the *Salmonella* YhjJ on the host, we found with the *in silico* interaction prediction approach that MAP1LC3B/LC3B can bind to YhjJ, potentially as a self-defense mechanism (Figure 3(c)).

**Figure 4.**
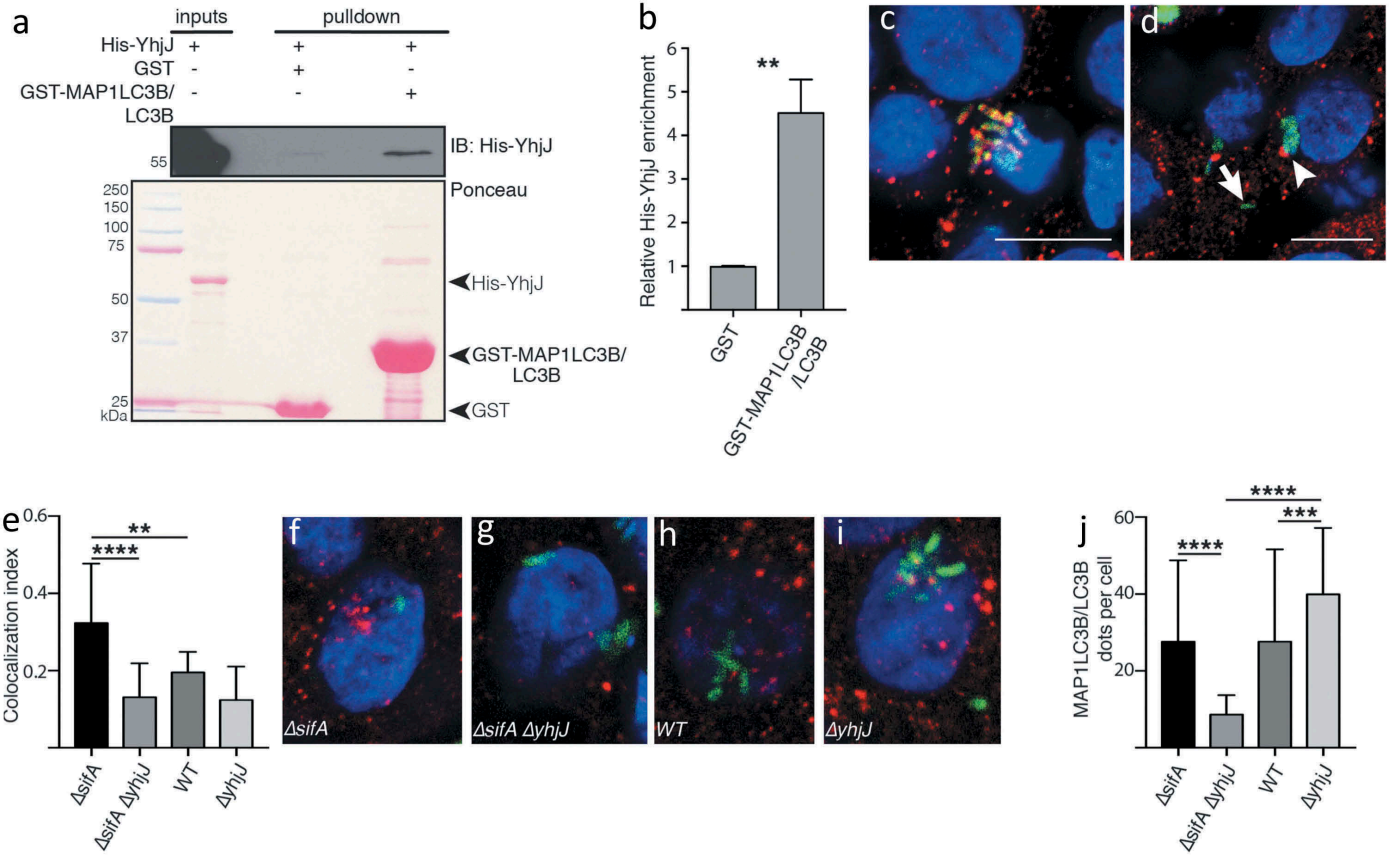
*Salmonella* YhjJ protease interacts with MAP1LC3B/LC3B (**a**) GST affinity-isolation assay between recombinant GST-MAP1LC3B/LC3B and His-YhjJ. Upper panel: immunoblot against 6xhistine-tagged YhjJ; lower panel: Ponceau S staining. (**b**) Quantification of the enrichment of His-YhjJ based on 3 independent replicates. (**c, d**) Illustration of events of complete co-localization (**c**) adjacent localization (**d**, arrowhead) or no co-localization (d, arrow) between GFP-tagged *S*. Typhimurium (green) and MAP1LC3B/LC3B (red). Nuclei are stained with DAPI (blue). Scale bars: 10 µm. (**e**) Quantification of the ‘co-localization index’ (see Materials and Methods section). (**f-i**) Representative single HT-29 cell pictures from cells infected with ∆*sifA* (**f**) ∆*sifA*∆*yhjJ* (**g**) wild type (**h**) or ∆*yhjJ S*. Typhimurium (**i**). (**j**) Quantification of the number of MAP1LC3B/LC3B dots per individual cell. Bar charts show mean ± s.d. Statistical significance was determined using Students’ t-test (b) or one-way ANOVA (e, j), **P < 0.01, ***P < 0.001, ****P < 0.0001. For a full description of the statistics, refer to Table S8.

To validate whether the *Salmonella* YhjJ protease via its LIR motif (Figure S1) indeed interacts with MAP1LC3B/LC3B, we performed a GST affinity-isolation assay between recombinant GST-MAP1LC3B/LC3B and 6xHis-YhjJ from *Salmonella* Typhimurium expressed in *E. coli*. We observed that 6xHis-YhjJ is significantly enriched in the affinity isolation with GST- MAP1LC3B/LC3B compare to GST alone (Figure 4(a,b); Table S8), thus confirming that MAP1LC3B/LC3B binds YhjJ. To confirm the functional importance of YhjJ protease in autophagy modulation, we infected HT-29 human epithelial cells with various bacterial strains derived from *S. Typhimurium* SL1344 for 6 h. As expected, the deletion of the *sifA* gene increased the frequency at which *S*. Typhimurium escapes the intracellular *Salmonella*-containing vacuole (SCV), hence raising the likelihood of the secreted YhjJ to encounter the autophagy machinery and reflecting what normally happens to up to 20% intracellular WT *Salmonella* cells during infection of epithelial cells [64,65]. In the absence of YhjJ in the cytosolic *Salmonella* strain (TK0024, ∆*sifA*∆*yhjJ*), significantly fewer *Salmonella* cells colocalized with MAP1LC3B/LC3B compared with a single Δ*sifA* mutant (TK0019), suggesting that YhjJ is affecting autophagy (Figure 4(c-e); Table S8). Furthermore, the reduction of MAP1LC3B/LC3B recruitment to the bacteria was correlated with a significant decrease in MAP1LC3B/LC3B-positive structures per cell (Figure 4(f-j); Table S8), and we also observed an increase in SQSTM1/p62-positive structures (Figure S2) confirming YhjJ interferes with autophagy regulation when *Salmonella* is in the host cell cytosol.

Similar example is the C5a-peptidase (ScpA), whose orthologs are present in 3 different strains of *Streptococcal pyogenes*, and known to be required to establish infection and promote virulence in mice [66]. Using domain-domain and domain-motif predictions, we predicted that the C5a-peptidase *via* its Subtilase peptidase-domain (PF0082) could potentially cleave several core autophagy proteins including ATG5, which has been reported to be essential for host cells to defend themselves from *Streptococcal* infections [67]. We also predicted that C5a-peptidase could putatively be targeted by CALCOCO2/NDP52, suggesting that the host autophagy machinery could target this particular bacterial protein involved in autophagy modulation (Figure S3).

These and several other interplays (listed in Table S7) could serve as potential examples for the complex evolutionary arms race between virulence-associated traits of bacterial pathogens and host defense mechanisms, in particular autophagy based bacterial targeting.

### Conclusion

We present here an interaction network analysis on the complex interplay between bacterial proteins and the autophagy machinery, highlighting potential ubiquitin independent bi-directional connections. We showed that host autophagy proteins could be capable to recognize genus-specific bacterial proteins, indicating that they could potentially have evolved to target specific bacterial proteins. The 3 autophagy targeting proteins we examined have very little overlap for their bacterial protein targets but they often overlap in targeting the same bacterial genus. Still, there are a number of bacterial pathogens that are probably recognized only by a specific autophagy protein. For the cases of these interactions, if the given autophagy protein is mutated, the host could be more susceptible to chronic disorders associated with infection (such as in Crohn disease or ALS). As virulence factors were shown to be mostly targeted by CALCOCO2/NDP52 and MAP1LC3/LC3 but not SQSTM1/p62, this suggests that SQSTM1/p62 preferably and potentially targets generic bacterial proteins, and that CALCOCO2/NDP52 and MAP1LC3/LC3 evolved to target those specific pathogenic virulence proteins that SQSTM1/p62 is unable to recognize. To offset any biases caused by our selection criteria focusing only on pathogens and the proteins thereof selected on the basis of their spatial localization and access to the host machinery, our control analysis performed using outer membrane vesicle-derived proteins from enterotoxigenic (pathogenic) and non-toxigenic (non-pathogenic) strains of *Bacillus fragilis* suggest that autophagy has a greater propensity to target pathogenic proteins than non-pathogenic ones. Pathogens are known to modulate various phases of autophagy as a defense mechanism and for their own survival. Interestingly, some autophagy phases are modulated specifically by only a few pathogens, while other phases, such as ‘cargo recognition and packaging’ are modulated by multiple genera, highlighting both the genus-specific relevance of certain autophagy phases and the general importance of core autophagy processes, respectively. We propose this mechanism as a potential evolutionary arms-race since some of the bacterial proteins that could potentially modulate autophagy were also targeted by autophagy proteins as a self-defense mechanism for the host. In vitro testing with the *Salmonella* Typhimurium YhjJ zinc protease, known to be produced and secreted during infection, validated the robustness of our computational pipeline. We confirmed that MAP1LC3B/LC3B can bind in vitro YhjJ. In the wt background, upon infection, most Salmonella reside in SCVs. However, a small fraction of Salmonella escape from the SCVs to the host cell cytosol where they hyperproliferate [68] and disseminate to new hosts. We speculate that this cytosolic fraction of Salmonella secretes YhjJ protease that potentially cleaves ATG proteins (as we predicted) and inhibits autophagy. Therefore, in the wild-type background, ∆yhjJ mutants would exhibit increased autophagy. On the other hand, in ∆sifA mutants, most of Salmonella are cytosolic and this could trigger autophagy massively. In this case, we speculate that YhjJ via its interaction with MAP1LC3B/LC3B, may facilitate autophagic degradation of Salmonella, therefore reduced anti-Salmonella autophagy is observed in *sifA/yhjJ* double mutants. Therefore, this dual proposed role of YhjJ in autophagy may rely on the proportion of Salmonella bacteria in the cytosol. Overall, our systems-level analysis has highlighted the complex interplay between host autophagy and bacteria to inspire future experimental studies to elucidate the detailed molecular mechanisms of autophagy in the pathogenesis of bacterial infections.

Recognition of bacterial cargoes by the various autophagy receptors involves a number of complex interactions based on various post-translational signatures, the most common of which is ubiquitination. The bi-directional ubiquitome has been characterized on a systemic basis within the context of infections by particular pathogens such as *Salmonella* [34]. We complemented this approach with a systemic and computational analysis of ubiquitin independent mechanisms driving bacterial proteins targeted by autophagy receptors via recognition motifs, and autophagy proteins modulated by interactions with pathogen proteins. As we were able to correlate systemic patterns with rational biological explanations, we could demonstrate which interactions are unlikely to occur, at least through direct protein-protein interactions. Thus, in this context, although our approach does not cover ubiquitination mediated autophagy or autophagy receptors other than SQSTM1/p62 and CALCOCO2/NDP52, the identified putative targets in our study provide a novel insight for the range of substrates for the 2 autophagy receptors studied herein. We have to note however, that these proteins and others not identified as putative targets could still either be possible targets of ubiquitination enzymes, which mark their substrates for ubiquitin-dependent autophagic clearance mechanisms, or for other autophagy receptors like NBR1 [69].

Because autophagy is a major upstream modulator of the inflammasome, the findings we present here could shed new light on how pathogens indirectly evade the immune system by subverting autophagy. Our observations also provide plausible mechanistic explanations for increased infection susceptibility related with disorders such as Crohn disease. Validation results emanating from this study will potentially boost the development of novel pharmacological agents such as immunomodulators and autophagy modulating drugs which will be especially important in the current era of antibiotic resistance [70].

## Materials and methods

### Proteomes and inferring protein localizations

We analyzed 56 pathogenic bacterial species (Table S1) from 26 different genera with known associations to autophagy based on literature evidence. The proteomes of the selected organisms were retrieved from Uniprot (accessed May 05 2016). The bacterial proteins were filtered for their spatial accessibility (for the autophagy receptors to target the bacterial proteins and for them in turn to be able to physically interact with the core autophagy proteins via their corresponding motifs) by exploiting the cellular localization profiles. Only secreted, extracellular, outer-membrane and surface proteins were included by integrating protein annotation information from the Gene Ontology cellular localization terms, already existing cellular localization profiles and transmembrane and signal-peptide predictions retrieved from Uniprot [71]. In addition to the annotation based profiling, de novo localization prediction was performed using the PSORTdb standalone prediction software [72] in order to assign localization for proteins without existing localization annotations. For all further analysis, only the bacterial proteins shortlisted after the spatial filtering process were used. Information on virulence factor annotation and protein classification into the various virulence factor categories was retrieved from the VFDB resource [47].

### Protein-protein interaction predictions

The consensus binding motifs of the autophagy receptors SQSTM1/p62 [73] and CALCOCO2/NDP52 [74] were used to scan the bacterial proteins in order to predict their putative targets. The presence of functional LIR motifs in bacterial proteins was examined using the iLIR software (https://ilir.warwick.ac.uk). The predictions for MAP1LC3/LC3 interacting proteins is based on the presence of putative functional canonical LIR motifs [75,76]. The predicted LIR motif-containing proteins have been shown to interact with all Atg8-family proteins in human (MAP1LC3A/LC3A, MAP1LC3B/LC3B, MAP1LC3/LC3C, GABARAP, GABARAPL1, GABARAPL2). However, since MAP1LC3C/LC3C has been shown to interact with non-canonical LIR motifs [12], iLIR predictions do not apply for MAP1LC3C/LC3C or any interaction that depends on a non-canonical LIR motif. iLIR uses structural filtering based on disordered region inferences to filter the LIR motifs thus yielding the putative MAP1LC3/LC3 targets. These ‘disordered regions’ are stretches of residues in the protein sequence which do not correspond to a well-defined three-dimensional structure but are still functional. These regions are generally characterized by the lack of hydrophobic amino acids which make up hydrophobic cores and structured domains and they expose short 3–10 residue stretches of amino acids called motifs which mediate the interaction with other protein domains. By virtue of these properties, disordered regions actively participate in a range of protein functions and are often subjected to post-translational modifications, thus broadening the number of functional states in which the protein can exist within the cell [77].

Domain-domain and domain-motif prediction tools as described elsewhere [78,79] were used to identify bacterial proteins which have the potential to interact with and modulate host core autophagy proteins which also includes the selective autophagy receptor SQSTM1/p62 and the autophagy adaptor protein MAP1LC3/LC3. These methods enable the detection of potential post-translational modifications mediated between domains and motifs present in the microbial and host proteins. The reference set of all known and validated domain-domain and domain-motif pairs were retrieved from the DOMINE [80] and ELM [81] databases. In brief, if a bacterial protein harbors a domain which is already known to interact (from the gold set) with a domain or motif within a host autophagy protein, then that particular bacterial protein is considered to post-translationally modulate the host autophagy protein. The bacterial proteins described were derived from the earlier spatial filtering process. The list of core autophagy proteins was retrieved from the Autophagy Regulatory Network resource (http://autophagyregulation.org) [82]. Information about the stage-wise classification of autophagy proteins was retrieved from [83]. Orthology grouping was performed using InParanoid [84].

The 2 sets of predictions (bacterial proteins which are capable of being targeted by host autophagy receptors or MAP1LC3 and the bacterial proteins which could potentially modulate the activity of host autophagy proteins) described above were annotated based on the structural features such as disordered regions and globular domains [77] of the proteins involved. We then utilized information on disordered regions to highlight the probable motifs that could be modified and accessed by the domains of other proteins. Disordered region predictions for the proteins of interest were generated using the standalone version of IUPRED [85,86]. We used the InterProScan tool [87] to obtain *de novo* domain annotation for the proteins of interest. All custom codes for the analysis were written in Python.

Interactions for the experimental validation were shortlisted based on several criteria: (1) the bacterial protein should be involved in bi-directional interactions – in other words, the bacterial protein needs to be a potential target for 1 of the autophagy receptors and/or MAP1LC3/LC3 and be predicted to interact and post-translationally modify host core autophagy proteins; (2) in the case of MAP1LC3/LC3 targets, the bacterial protein needs to have an iLIR score of at least 18 as a higher iLIR score indicates increased significance of the predicted target; (3) the corresponding motifs (involved in the bi-directional interactions) to be within disordered regions; (4) experimental evidence for expression of the bacterial protein under infection conditions; (5) experimental evidence for the pathogenic protein to be in the bacterial secretome.

### Cloning *yhjJ* in pET28a(+)

The *yhjJ* gene, encoding an M16 zinc protease, from *Salmonella* Typhimurium was produced by PCR amplification from gDNA from *S*. Typhimurium strain SL1344. To clone *yhjJ* into pET28a(+) plasmid (Novagen, Merck Millipore, 69,864), we designed the the yhjJ_F and yhjJ_R forward and reverse PCR primers (Table 1.docx). The forward primer incorporates the *Bam*HI restriction site, while the reverse primer includes *Not*I site (underlined). The reverse primer also includes a TAA stop codon before the restriction site. The PCR product was cloned into pET28a(+) vector using *Bam*HI and *Not*I enzymes and subsequent ligation. The resulting pET28-YhjJ plasmid was checked by sequencing. The resulting plasmid constructs were cloned into NEB® 10-beta Competent *E. coli* cells (New England Biolabs, C3019).

**Table 1.** Oligonucleotides used in this study.

Name	Sequence	Use in this study
3578delF	5ʹ-GCTGTCTTTTTATTACCAGGATTGTTGATCAGGGGTTCACgtgtaggctggagctgcttc-3ʹ^a^	Construction of gene deletion mutant
3578delR	5ʹ-GCCCGGTGGCGCTGCGCTTACCGGGCCGACAGGCGGCAGCcatatgaatatcctccttag-3ʹ^a^	Construction of gene deletion mutant
sifA_RedF2	5ʹ- ATTATGTAGTCATTTTTACTCCAGTATAAGTGAGATTAATcatatgaatatcctccttag-3ʹ^a^	Construction of gene deletion mutant
sifA_RedR2	5ʹ- TAAACCCTGAACGTGACGTCTGAGAAAGCGTCGTCTGATTGt gtaggctggagctgcttc-3ʹ^a^	Construction of gene deletion mutant

^a^ Uppercase sequences indicate homology with the flanking regions of the target gene

### Protein expression and GST affinity isolation

pET28a-YhjJ, pGEX-4T1 (GE Healthcare Life Sciences, 28954549) and pGEX-4T1- MAP1LC3B/LC3B (gift from Dr Rob Layfield, School of Life Sciences, University of Nottingham) were transformed into Rosetta2(DE3) pLacI competent cells (Novagen, Merck Millipore 71404) that were grown on plates supplemented with kanamycin and chloramphenicol (pET28a-YhjJ) or ampicillin and chloramphenicol (pGEX-4T1 and pGEX-4T1-MAP1LC3B/LC3B). For expression, overnight cultures were diluted 1:100 in fresh LB medium, grown to OD_600_ = 0.6 at 37ºC, and induced with 0.5 mM Isopropyl β-D-1-thiogalactopyranoside. Cells were harvested by centrifugation after 3 h at 37ºC (pET28a-YhjJ) or 16 h at 2ºC (pGEX-4T1 and pGEX-4T1- MAP1LC3B/LC3B), and lysed by sonication in Classic Lysis Buffer (CLB; 25 mM Tris, pH 7.4, 100 mM NaCl, 2 mM EDTA, 0.05% *beta-*mercaptoethanol supplemented with a protease inhibitor cocktail (Roche, 5892791001). Following clarification of the lysates by centrifugation at 14310.4 g for 20 min at 4ºC (Beckman centrifuge, JA-20 rotor), GST-fusion proteins were purified on glutathione-Sepharose 4 Fast Flow beads (GE Healthcare, 17–5132-01) for 30 min at 4ºC. Beads were rinsed with at least 10 beads volumes of High Salt Wash Buffer (25 mM Tris, pH 7.4, 500 mM NaCl, 2 mM EDTA) and Low Salt Wash Buffer (25 mM Tris, pH 7.4, 50 mM NaCl, 2 mM EDTA) before incubation 6xHis-YhjJ for 2 h at 4ºC. The beads were washed 4 times with CLB supplemented with 10 mM imidazole and boiled in 2X Laemmli loading buffer for 5 min.

Cell lysates and GST affinity-isolation eluates were subjected to SDS-PAGE (10% polyacrylamide gel) followed by transfer onto nitrocellulose membrane for 60 min at 100 V. Membranes were stained with Ponceau Red (Sigma-Aldrich, P3504) before blocking with 5% non-fat milk in TBST (0.1% Tween-20 [Sigma-Aldrich, P9416] in TBS (Tris-buffered saline; 20 mM Tris and 150 mM NaCl) for 1 h. Primary antibody anti-His tag (Abcam, ab18184; 1:5,000) was incubated for 2 h in TBST, followed by incubation with HRP-coupled secondary goat anti-rabbit antibody (Thermo Scientifics, NE171565) for 45 min in TBST+1% non-fat milk. All antibodies incubations were performed at room temperature with gentle agitation. The membrane were developed by chemiluminescence (Amersham ECL Reagents; GE Healthcare Life Sciences, RPN2134).

### Bacterial strains and growth conditions

The *S. typhimurium* SL1344 (initially obtained from Catherine Lee [88]) and JH3009 strains used in this study were kindly provided by Jay Hinton [89]. All strains are listed below in Table 2. Strains were grown in 25 ml of LB broth [90] at 37°C, unless stated otherwise. Cultures were shaken in 250-ml conical flasks at 2.23 g. For invasion assays, a 1 in 100 dilution of a 5 ml overnight bacterial culture was grown in 25 ml of LBS (LB containing a total of 0.3 M NaCl) in 250-ml conical flasks until an optical density at 600 nm (A600) of 1.2. Antibiotics were added as required at the following final concentrations (ampicillin, 100 mg ml-1; kanamycin, 50 mg ml-1; chloramphenicol, 10 mg ml-1).

**Table 2.** Bacterial strains used in this study.

Strains	Description	Source or Reference
SL1344	4/74 *hisG rpsL*	Ref [88]
JH3009	SL1344 ɸ(*ssaG’-gfp^+^*), Cm^R^	Ref [89]
TK0016	JH3009 ɸ(*ssaG’-gfp^+^*), Δ*yhjJ*, Km^R^, Cm^R*^	This study
TK0017	SL1344 Δ*yhjJ*, Km^R^	This study
TK0018	SL1344 Δ*sifA*, Km^R^	This study
TK0019	JH3009 ɸ(*ssaG’-gfp^+^*), Δ*sifA*, Km^R^, Cm^R*^	This study
TK0021	SL1344 Δ*sifA*, Km^S^	This study
TK0024	JH3009 ɸ(*ssaG’-gfp^+^*), Δ*sifA* Δ*yhjJ*, Km^R^, Cm^R*^	This study

* ɸ indicates transcriptional fusion. The ɸ*ssaG’-gfp^+^* fusion is inserted in the *putPA* chromosomal locus as described before [89].

### Bacterial gene deletion and replacement

The *yhjJ* and *sifA* gene coding sequences were independently deleted from the *S*. Typhimurium strain SL1344 chromosome. Briefly, for each deletion a kanamycin resistance determinant was amplified by PCR from the pKD4 plasmid template using primers listed in Table 1 [91]. Each of these primers includes at its 5′ ends a 40 base-long extension showing homology with the flanking regions of the respective target gene. The generated PCR products were used to replace the structural target genes on *S. Typhimurium* chromosome using the Lambda Red recombination system [91]. The TK0017 and TK0018 recombinant strains generated were selected for antibiotic resistance, verified by analytical PCR and transduced back into a clean SL1344 background using P22 phage transduction to avoid possible non-intended recombination events [92] (Table 2).

The kanamycin resistance cassette replacing the *sifA* gene in TK0018 was excised from the chromosome using the yeast Flp recombinase expressed from the thermosensitive replicon pCP20 [93] generating the strain TK0021. The pCP20 replicon was subsequently removed from TK0021 after culture at 40°C in non-selective medium. The *yhjJ*, Km^R^ deletion and the ɸ(*ssaG’-gfp^+^*), Cm^R^ transcriptional fusion were transduced into TK0021 by P22 phage transduction, generating the strain TK0024 used in this study (Table 2).

### Epithelial cell invasion assay

HT-29 intestinal epithelial cells (ATCC®, HTB-38™) were cultivated in complete medium composed of DMEM (Lonza, BE12-614F) supplemented with 10% heat-inactivated fetal bovine serum (Labtech, FCS-SA) and 2 mM L‑glutamine (Lonza, BE17-605E) in a humidified incubator at 37°C, 5% CO_2_. Between 2 and 5 × 10^5^ cells were seeded on glass coverslips (VWR, 631–0149; 13 mm diameter; Thickness No1) in 24-well plates. On the day of the invasion assay, cells were washed twice in non-supplemented DMEM and infected with bacterial suspensions in DMEM prepared from the LBS subculture of the *Salmonella* strains (see Bacterial strains and growth conditions) at a multiplicity of infection (MOI) of 10 bacterial cells per mammalian cell. Infected cells were incubated at 37°C, 5% CO_2_ for 30 min. The *Salmonella*‑containing medium was then replaced with complete medium containing 30 µg/ml gentamicin for 30 min to kill all *Salmonella* cells remaining extracellular. The gentamicin concentration was subsequently reduced to 5 µg/ml for the rest of the experiment. At 6 h post-infection, the medium was removed, cells were washed twice in Dubelcco’s phosphate-buffered saline (DPBS; Sigma Aldrich, D8537), fixed in 4% paraformaldehyde, at room temperature for 20 min, and washed twice for 5 min at room temperature in PBS prior to immunofluorescence labelling.

### Immunocytochemistry

For the MAP1LC3B/LC3B immunostaining, cells were first treated with 50 mM NH_4_Cl in PBS for 10 min, permeabilized in methanol for 5 min and washed 3 times in PBS for 5 min. Blocking was performed in 1% bovine serum albumin (BSA) Fraction V (Sigma‑Aldrich, 05479) in PBS for 30 min at room temperature. The rabbit anti- MAP1LC3B/LC3B antibody (Abcam, ab48394) and FITC-conjugated anti-GFP (Abcam, ab6662) applied were used at 1:2000 and at 1:200 in PBS containing 1% BSA fraction V (Sigma Aldrich, 05479), overnight at 4°C, respectively. For SQSTM1/p62 immunostaining, fixed cells were permeabilized and blocked in 1% BSA, 0.1% saponin (Fluka, 84,510) in PBS for 30 min at room temperature. The rabbit anti-SQSTM1/p62 antibody (Abcam, ab91526) was used at 1:6000 in PBS containing 1% BSA, 0.1% Saponin, overnight at 4°C. All primary antibodies were washed 3 times in either 1% BSA, PBS (MAP1LC3B/LC3B) or 1% BSA, 0.1% saponin, PBS (SQSTM1/p62). Alexa Fluor 594‑conjugated anti-rabbit secondary IgG (Abcam, ab91526) was applied at 1:1000 dilution and DAPI stain at 1:2000 in the respective buffer for each primary antibody for 1 h at room temperature. Coverslips were then washed 3 times in respective buffers, once in water and mounted using Fluoromount‑G anti-fading compound (ThermoFisher Scientific, 00‑4958‑02). Coverslips were left to set, sealed using nail varnish and stored at ‑4°C until observation. Slides were imaged on a Zeiss LSM710 microscope, using a 100x Apochromat (100x/1.4 Oil DIC plan Apo) oil immersion objective. Imaged areas were chosen randomly based solely on the DAPI staining to avoid unconscious-bias.

### Image analysis

For the semi-automated image analysis for SQSTM1/p62 and MAP1LC3/LC3 stainings, ImageJ/Fiji was used for image analysis. Individual cells, identified by the DAPI staining of their nuclei, were segmented, and objects on the edges and segmentation artefacts (small objects) were removed. Individual cell masks (ROIs) were saved for each image. For SQSTM1/p62 staining, an automated macro was used to collect the mean signal intensity data for all 3 channels and area data was gathered for every single segmented cell and saved into an Excel spreadsheet. For the analysis of MAP1LC3/LC3 puncta per cell, only the cells containing at least 1 bacteria were used (manual curation). An automated macro was used to identify the MAP1LC3/LC3 puncta (autophagosomal structures). Information related to the number of dots per cells were gathered for every single segmented cell and saved into an Excel spreadsheet. Macros are available upon request.

### Quantification of LC3-Salmonella colocalization

For the quantification of colocalization between *Salmonella* and MAP1LC3/LC3, we used a co-localization index (CI) calculated using the following formula:

$$CI\;=\;\;\frac{1\times n(coloc)\;+\;0.5\times n(adjacent)\;+\;0\times n(noLC3)}{n(total)}$$

As such, n(coloc) corresponds to the number of bacteria that colocalize completely with LC3 and was attributed the value 1, n(adjacent) corresponds to the number of bacteria that are immediately adjacent/touching MAP1LC3/LC3 structure and was given the value 0.5, and n(no LC3) correspond to the bacteria that are neither co-localizing or close to MAP1LC3/LC3 structures and was given a null value.

### Statistical analyses

Statistical analyses were done with Prism7/8 software (GraphPad). D’Agostino & Pearson normality test was used to test the normality of the sample distribution. Two-tailed t-test was used for the comparison of 2 groups. To compare 3 or more groups, one-way ANOVA was used. For the comparison of more than 3 groups, the multiple comparisons were corrected using Sidak’s or Dunn’s Multiple comparison test. Dunn’s test was done on data that do not meet the assumption of parametric test (normal distribution).
