# Supplementary figures and images for: Targeted interplay between bacterial pathogens and host autophagy
Source: Autophagy. 2019 Mar 25;15(9):1620–33. doi: 10.1080/15548627.2019.1590519 (PMC6693458; doi:10.1080/15548627.2019.1590519)

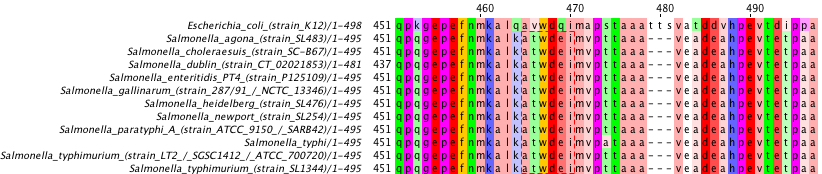

Supplement: Supplemental Material [file kaup-15-09-1590519-s001.zip › Supplementary information/2017AUTO0330R7-s02.jpg]
