# Supplementary material for: Targeted interplay between bacterial pathogens and host autophagy
Source: Autophagy. 2019 Mar 25;15(9):1620–33. doi: 10.1080/15548627.2019.1590519 (PMC6693458; doi:10.1080/15548627.2019.1590519)
Supplement: Supplemental Material [file kaup-15-09-1590519-s001.zip › Supplementary information/2017AUTO0330R7-s01.docx]

Norwich, UK

21^st^ February 2019

Dear Dr. Klionsky,

Thank you for your email on the 18th of February 2019 with the final comments regarding our manuscript (2017AUTO0330, Sudhakar et al, "Targeted interplay between bacterial pathogens and host autophagy").

We have accepted the changes you suggested and added the necessary information where requested including the new methods section on statistical analyses and details of reagents. In addition, we have corrected other instances of the typos based on your suggestions.

We would like to thank you for providing the feedbacks.

I have uploaded the revised manuscript to the website of Autophagy.

Thank you for your support in the whole review process and for accepting the manuscript for publication in Autophagy.

Sincerely yours,

Tamás Korcsmáros Ph.D.

Computational Biology Fellow

Group leader
